# Neural masses and fields in dynamic causal modeling

**DOI:** 10.3389/fncom.2013.00057

**Published:** 2013-05-28

**Authors:** Rosalyn Moran, Dimitris A. Pinotsis, Karl Friston

**Affiliations:** ^1^Wellcome Trust Centre for Neuroimaging, Institute of Neurology, University College LondonLondon, UK; ^2^Virginia Tech Carilion Research Institute, Virginia TechRoanoke, VA, USA; ^3^Bradley Department of Electrical and Computer Engineering, Virginia TechBlacksburg, VA, USA

**Keywords:** dynamic causal modeling, electroencephalography, magnetoencephalography (MEG), local field potential (LFP), neural mass models

## Abstract

Dynamic causal modeling (DCM) provides a framework for the analysis of effective connectivity among neuronal subpopulations that subtend invasive (electrocorticograms and local field potentials) and non-invasive (electroencephalography and magnetoencephalography) electrophysiological responses. This paper reviews the suite of neuronal population models including neural masses, fields and conductance-based models that are used in DCM. These models are expressed in terms of sets of differential equations that allow one to model the synaptic underpinnings of connectivity. We describe early developments using neural mass models, where convolution-based dynamics are used to generate responses in laminar-specific populations of excitatory and inhibitory cells. We show that these models, though resting on only two simple transforms, can recapitulate the characteristics of both evoked and spectral responses observed empirically. Using an identical neuronal architecture, we show that a set of conductance based models—that consider the dynamics of specific ion-channels—present a richer space of responses; owing to non-linear interactions between conductances and membrane potentials. We propose that conductance-based models may be more appropriate when spectra present with multiple resonances. Finally, we outline a third class of models, where each neuronal subpopulation is treated as a field; in other words, as a manifold on the cortical surface. By explicitly accounting for the spatial propagation of cortical activity through partial differential equations (PDEs), we show that the topology of connectivity—through local lateral interactions among cortical layers—may be inferred, even in the absence of spatially resolved data. We also show that these models allow for a detailed analysis of structure–function relationships in the cortex. Our review highlights the relationship among these models and how the hypothesis asked of empirical data suggests an appropriate model class.

## Introduction

Over the past two decades, BOLD neuroimaging techniques have been successfully applied in human studies to identify regions of functional specialization, to within a scale of a few millimeters (Ashburner, 2012). Electrophysiological recordings including magneto- and electro-encephalography (M/EEG) offer an aggregate measure of neuronal activity (in the order of hundreds of thousands of neurons) but at a millisecond timescale (Baillet et al., 2001; Nunez and Srinivasan, 2006). Though localizing activity is mathematically ill-posed in these electromagnetic modalities, the wealth of spatial information from fMRI studies can now support M/EEG as a powerful modality for the analysis of functional integration in the human brain.

Dynamic causal modeling (DCM) is designed to probe the mechanisms of effective connectivity (the influence of one brain region on another) that underlie multi-region network responses in neuroimaging (fMRI, M/EEG) data. The approach uses a neurobiologically motivated model which is inverted or fitted to empirical observations using Bayesian techniques (Daunizeau et al., 2011). These comprise separate generative processes at the neuronal level and at the observation level. For M/EEG, neural mass and neural field models in particular are used, to support this analysis by quantifying the temporal and spatiotemporal evolution of macroscopic brain activity, using physiologically plausible dynamics. In DCM; as implemented in the SPM software (Litvak et al., 2011), neural mass and field models are used as generative models to infer the synaptic parameters and effective connectivity that constitute active brain networks. More recently, these models have been applied in (single-region) DCMs as a “mathematical microscope”—to test synaptic hypotheses at the level of specific laminae and receptors (Moran et al., 2011b), and disambiguate between structural and functional hypotheses; for example, explaining intersubject variations in gamma oscillations (Pinotsis et al., 2013b). In this review, we summarize the state-of-the-art in modeling such population-based activity and demonstrate their use in the context of DCM. The development of these modeling approaches has been underpinned by pioneering developments several decades ago that produced generative models of EEG data characteristics based on neural masses (Wilson and Cowan, 1972; Nunez, 1974; Freeman, 1975, 1987; Jansen and Rit, 1995; Valdes et al., 1999; Wendling et al., 2000). These models have since been refined and extended (Wright and Liley, 1996; Rennie et al., 2000) to examine a myriad of neurobiological processes including anesthesia (Steyn-Ross et al., 1999), epilepsy (Breakspear et al., 2006; Marten et al., 2009; Nevado-Holgado et al., 2012), “resting state” brain dynamics (Deco and Jirsa, 2012), and so on. The types of models we will review here have played a direct implementational role in DCM and do not represent an exhaustive overview—a non DCM-centric treatment is provided in Deco et al. (2008). Here we consider, in particular, how DCM treats ensemble neuronal activity as a point process (neural mass models) or explicitly incorporate a spatial dimension (neural field models). Both types describe so-called “mesoscopic” properties of neural activity, employing statistical mechanics to transform single unit activity into population activity—where appropriate composites can be used to generate macroscopic data. Since this mesoscale is hidden from direct observation, we demonstrate how these models rely upon and exploit knowledge about synaptic and cell physiology, as well as neuroanatomy.

Two distinct biological perspectives have informed the development of neural models, leading to a taxonomy of “convolution” or “conductance-based” models. These distinctions arose from the consideration of cortical mesocolumns—convolution models (Freeman, 1975); and separately from the consideration of a single cell's electrophysiological properties—conductance models (Hodgkin and Huxley, 1952). Early work by Wilson and Cowan (1973) derived a sigmoidal relationship for transforming population membrane potential to an average population firing rate. These models consisted of sigmoidal and convolution-based operators and were refined on the basis of empirical observations by Freeman, Wendling and others (Freeman, 1987; Jansen and Rit, 1995; Wendling et al., 2000). In contrast, conductance based models were formulated as an equivalent circuit model of an excitable cell membrane: Hodgkin–Huxley's original description of the giant squid axon is the classical example of this sort of model—and was reduced to a two dimensional form by Morris and Lecar (1981). Their reduced circuit has been scaled up for M/EEG analysis in DCM using the Fokker-Planck formalism (Breakspear et al., 2010) to describe the evolution of population densities (Marreiros et al., 2009). In this setting, when only the first order statistics (e.g., mean) of the population density are considered, the model describes a neural mass (where the population density can be regarded as a point of mass). When higher order statistics are considered, we obtain a “mean-field” model (where the full density of one population depends on the mean of another). We note that the terminology here is rather specific to DCM; the focus of this review. In other settings, and in other treatments of neuronal activity (Coombes, 2010; Buice and Chow, 2013), mean-field models often refer to population dynamics with interacting means only. However, here we use the term *neural-mass* to refer to an interaction in population means and *mean-field* to higher-order interactions to remain consistent with the DCM literature and to acknowledge the early neural-mass nomenclature developed by Valdez-Sosa and other pioneering work in this field (Valdes et al., 1999). Both neural mass and mean field formulations can be applied to convolution and conductance based models: The choice of either convolution or conductance based model depends on the type of inference required (when applying the model to real data), with the latter offering a richer and biologically more realistic parameterization of synaptic currents.

The deployment of neural mass (or mean field) models of populations in DCM entails further neurobiological plausibility, through a laminar specification of cell types and their interconnectivity. For neocortical studies, a laminar architecture is populated with neuronal ensembles, so that forward (e.g., thalamo-cortical), backward or lateral (e.g., inter-hemispheric) extrinsic connections impinge upon pyramidal, spiny stellate or inhibitory interneurons (David et al., 2006). This construction is motivated by tracing studies in the macaque (Felleman and Van Essen, 1991) and demonstrates the first constraint under which these models were developed for DCM. Namely; that they conform to known physiological and anatomical principles. A second constraint is that they must be able to generate stereotypical features of empirical macroscopic measurements; for example, dominant alpha rhythms (David and Friston, 2003) or late potentials in evoked transients (Garrido et al., 2007a). In this sense, none of the models are “right” or “wrong”—but can be usefully compared to test a particular hypothesis (Box, 1976).

In addition to the distinction between neural mass and mean field formulations of either convolution or conductance based models, we also have to consider the distinction between models based upon ordinary differential equations and partial differential equations (PDEs) that endow neuronal populations with spatial attributes: incorporating the spatial domain into DCM was motivated by the advent of spatially resolved population recording modalities (Pinotsis et al., 2012). This use of neural fields, was proposed as a semi-quantitative treatment of electromagnetic brain activity by Jirsa and Haken (1996, 1997) and Robinson (2006). Crucially neural fields enable local axonal arborization to be modeled directly and can generate topological data features. These may be particularly resolved in high-density subdural grid electrodes (electrocorticography) and optical imaging techniques and also contribute to the topographical distribution of sensor/scalp space measurements in M/EEG.

In this review, we hope to provide a didactic treatment of the neural mass and neural field models available in DCM and highlight application studies that exemplify their use. This complements more general treatments of neural population modeling (Deco et al., 2008). The first section considers convolution-based neural mass models. We will demonstrate their use in inferring causal interactions among multiple brain regions and highlight the minimal assumptions needed to form—and test—competing hypotheses. In this section, we will also introduce the important distinction between different models and different data features; noting that the same models can be used for (and indeed should be capable of generating) different data features. We will focus on the distinction between time and frequency domain responses—highlighting the use of identical neural mass models when modeling evoked and steady state responses. In the second section, we examine conductance-based models and how new currents can be added to enhance physiological detail at the synaptic level. We also examine the impact of second-order interactions among neuronal ensembles in mean field models, particularly in the spectral domain. The third section introduces the spatial parameterization in the form of partial differential or neural field equations—and how these have been applied to test alternative explanations for gamma activity in the visual cortex. In this example, we reconsider lateral connections and the role of distinct pyramidal cell populations. This final model recapitulates “a canonical microcircuit” and provides a framework for investigating differences in directed oscillations. This development was motivated by theoretical considerations about message passing in the brain; namely predictive coding and implications for spectral asymmetries in laminar specific communication (Friston, 2005, 2009). Important asymmetries of the sort are evident in several recent empirical observations (Maier et al., 2010; Buffalo et al., 2011; Bastos et al., 2012) on the laminar specificity of oscillation frequencies. In principle, these sorts of observations can be used with DCM, to address key questions about reciprocal message passing in the brain and its hierarchical architecture.

## Convolution based neural mass model

### Generative models of evoked responses: the ERP model

**Figure 1:**
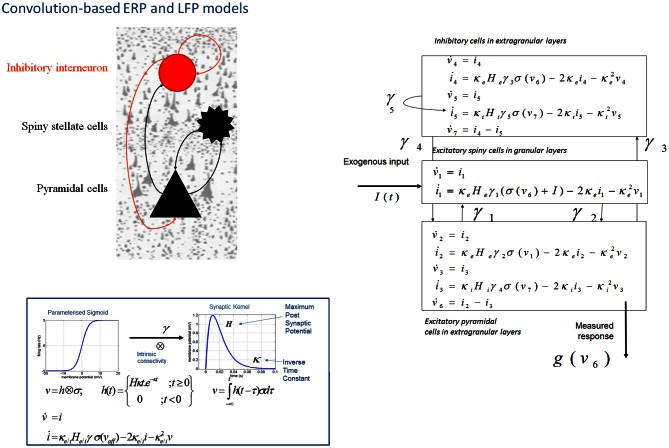
**Convolution-based neural mass models: “ERP” and “LFP”.** Neural mass model used to represent a cortical source. Three cell subpopulations contribute to the ongoing dynamics. These include spiny stellate cells in granular layer IV, pyramidal cells and inhibitory interneurons in extra granular layers (II and III; V and VI). Intrinsic connections, γ_1,2,3,4,5_ link subpopulations in each source. Neuronal states include currents, *i*, and membrane potentials *v*. Extrinsic connections enter at specific cortical layers (see main text). Right: The functions controlling ongoing dynamics and their parameterization are summarized by synaptic kernels, which are used to convolve presynaptic (firing) input [a sigmoidal function of presynaptic membrane depolarization σ(*v*)] to produce postsynaptic depolarization (*v*), dependent on membrane time constants (*1/*κ_*e*/*i*_) and average post-synaptic depolarizations (*H*_*e*/*i*_) at excitatory (*e*) and inhibitory (*i*) synapses.

In DCM, event related potentials are modeled as the response of a dynamic input–output system to exogenous (experimental) inputs (David et al., 2006; Kiebel et al., 2006; Garrido et al., 2007b). The DCM generates a predicted ERP as the response of a network of coupled sources to sensory (thalamic) input—with the form of a narrow (16 ms) Gaussian impulse function that accounts for some temporal smoothing in thalamic volleys. Each source is modeled as a point source (c.f., equivalent current dipole) comprising three subpopulations, each assigned to a particular cortical layer. For simplicity, we place an inhibitory subpopulation in the supragranular layer. This receives inputs from excitatory deep pyramidal cells in an infra-granular layer which are, in turn, driven by excitatory spiny cells in the granular layer; layer IV. These three subpopulations are connected with intrinsic coupling parameters as shown in Figure 1. Though these models operate as a point process, by specifying different layers we can call on anatomical rules of extrinsic (region to region) connectivity (Felleman and Van Essen, 1991). Specifically, the “ERP” and “LFP” convolution based models can be assembled within a generative network using three distinct types of connections. Forward connections correspond to afferent pyramidal axons and synapse on layer IV stellate cells, while backward afferents impinge upon pyramidal and inhibitory interneurons outside of layer IV. Lateral, inter-hemispheric connections are modeled with a postsynaptic response that is elicited in all layers.

The evolution of neuronal activity in this anatomical architecture is controlled by two simple operations following Jansen and Rit (1995). The first is a convolution operation (Figure 1) that lends the model its name and models the average membrane depolarization response as a low-pass impulse response. This transforms the average density of pre-synaptic firing arriving at the population into the average postsynaptic membrane potential (PSP). This response is specified by two biologically informed parameters; one tunes the maximum amplitude of PSPs and represents the receptor density and the second is a lumped representation of the sum of synaptic rate constants (of passive membrane and other spatially distributed delays in the dendritic tree). The output operator (Figure 1) then transforms this average membrane potential into the average rate of action potentials fired by the population. This transformation is assumed to be instantaneous and is described by a sigmoid function with parameters that determine its shape and location. These parameters model the voltage sensitivity or gain of the subpopulation and its average threshold. It is this function that endows the model with non-linear behaviors that are crucial for phenomena like phase-resetting of the M/EEG. The sigmoid form for these activation functions was originally motivated as arising from a unimodal distribution of threshold potentials within a population of Heaviside response units (Wilson and Cowan, 1973). More recent formulations that connect directly to full mean field (population density) treatments consider the sigmoid form to arise from the distribution of depolarizations within a population, under a fixed threshold. This output of this sigmoid function (presynaptic input) is scaled by intrinsic and extrinsic connectivity parameters from subpopulations within a source or from pyramidal cell afferents that arise from other sources in the network. Thus activity promulgates and reverberates throughout the network (Figure 1). Delays along these connections are also parameterized with values that correspond to the time taken for axonal propagation between layers (~2 ms) and regions (~16 ms).

A pair of ordinary differential equations completely describes the dynamics of each subpopulation within a source (Figure 1). These are deterministic and—for a DCM of ERPs—a thalamic impulse timed to correspond to some experimental stimulus perturbs the sources. The spatial arrangement of pyramidal cell dendrites (perpendicular to the cortical surface) renders them the prominent sources of measurable electromagnetic signals and are thus harvested from each source in the network and passed through a lead field, to produce the spatiotemporal patterns observed in M/EEG sensors.

Early applications of this model led to a series of EEG-based investigations into oddball effects by Garrido et al. (2007a, 2008, 2009). The mismatch negativity (MMN) is a negative change in the auditory evoked potential that occurs after an unpredictable change in the acoustic stream. It is elicited, for example, when deviant frequency tones are embedded in a stream of repeated tones. The MMN has a fronto-temporal topology and is thought to reflect the updating of an internal model of the sensorium, where by a sensory prediction error is registered and a new prediction formed (Näätänen et al., 2005). Competing “ERP” neural mass models were compared (in terms of their model evidence, using standard Bayesian techniques) to probe the type of extrinsic connection changes that mediate the MMN. Indicating both a bottom-up sensory prediction error and a top-down change in predictions, a stimulus specific modulation of both forward and backward connections among hierarchically deployed sources exhibited trial specific (deviant compared to standard) modulation (Garrido et al., 2007b). This network and paradigm has since been investigated in pathological settings. A striking example by Boly et al. (2011) used the same model comparison procedure to distinguish between top-down and bottom-up extrinsic connections and their changes with levels of consciousness.

### Generative models of evoked and spectral responses: the LFP model

A second convolution-based model, named the “LFP” model was developed from the “ERP” model. The model was augmented to address the neurotransmitter basis of changes in intracortical local field potentials from rat prefrontal cortex (Moran et al., 2007)—and now also serves as a generative model for non-invasive EEG and MEG studies (Boly et al., 2012). The model differed from the “ERP” model in two ways: first, based on biophysical models by Whittington et al. (1995) of gamma oscillations in the hippocampus, the role of inhibitory interneurons was augmented with recurrent self-connections (Figure 1). This subtle addition was important from the perspective of a new set of questions. Here our goal was to develop a generative model of spectral responses can exhibit high-frequency oscillations. A second extension refined the neurophysiological input-output transforms; whereby spike-rate adaptation was modeled at the input stellate cell population. This involves the addition of currents based on the phenomenological model described by Benda and Herz (2003) and combined several ionic currents modulating spike generation—including voltage-gated potassium currents (M-type currents), the interplay of calcium currents and intracellular calcium dynamics with calcium-gated potassium channels (AHP-type currents) and the slow recovery from inactivation of the fast sodium current.

Practically these additions lead to a larger dynamic repertoire using an identical connectivity architecture (three subpopulations within a source and extrinsic forward, backward and lateral connections with laminar specificity) as that described above. The focus of the LFP model was the reproduction of fast synchronous activity as summarized with the steady-state spectral density (Fourier transform) of time series data. In order to generate steady-state spectral responses, we linearized the model's differential equations around an equilibrium point. This equilibrium or operating point is obtained by integrating the system over a protracted time window. The linearity assumption will accommodate parameter spaces in the region of fixed points and local bifurcations (Friston et al., 2012), known to emerge from this sort of model (Grimbert and Faugeras, 2006). In DCM, the neural masses are treated as a system that is perturbed by white and pink noise; which is explicitly parameterized. This provides a compact summary of the system, where the system's spectral responses can be obtained from its transfer functions, which depend on the physiological parameters of the model (and neural noise) (Nunez, 1974; Steyn-Ross et al., 1999; Robinson et al., 2001; Moran et al., 2007). In other words, the transfer function links unobserved physiological processes to measured spectral responses and is an essential part of forward or generative models of spectral measures. This “LFP” neural mass model was first used in a single-region DCM analysis to demonstrate how one can make inferences about synaptic function at the neuronal level, using macroscopic electrophysiological measurements. This proof of principle used microdialysis measures of extracellular neurotransmitter for validation (Moran et al., 2008) and extended the breadth of applications of DCM—in this case by inferring condition specific modulations of synaptic parameters.

## Conductance based neural mass models

### The NMM and MFM

**Figure 2:**
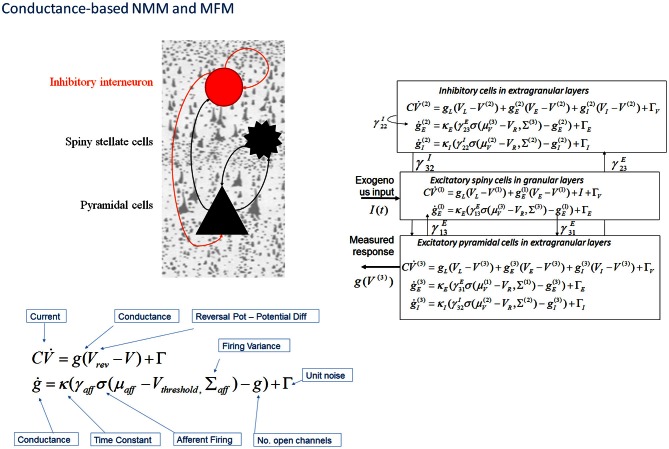
**Conductance-based neural mass models: “NMM” and “MFM”.** This figure shows Morris–Lecar-type differential equations describing the time evolution of a single cell current (capacitance × change of membrane potential: $C\dot{V}$) and conductance (*g*) at inhibitory interneurons (extra granular layers), spiny stellate cells (granular layers) and pyramidal cells (extra granular layers). In this model, all cell types possess AMPA receptors, GABA_A_; with ion-channel time constants (*1/*κ_*e*/*i*_). Layers are connected with strengths parameterized by γ V_L_, V_E_, and V_I_ are reversal potentials for leak potassium channels, sodium, and chloride channels, respectively, at V_T_ is the threshold potential. NMDA receptors at pyramidal cells and inhibitory interneurons can be added using a conductance equation of similar form, weighted by a voltage dependent switch (Moran et al., 2011a,b). For a full population Fokker-Planck characterization see Marreiros et al. (2008).

In DCM, the first biophysical model of ensemble activity to receive the three-letter acronym “NMM” was described in Marreiros et al. (2008, 2009). These models parameterize neuronal dynamics at the level of a single neuron and employ density-based flow (the time derivative of neural activity) mechanics to represent the dynamics of a population of neurons. In coupling multiple subpopulations with different characteristics within and between regions, the framework used a mean-field reduction (assumption); where each type of population comprises a probability density and is only affected by the main activity of other neuronal populations or ensembles in the model. This formal population treatment was applied in the context of a conductance-based model predicated on the Morris–Lecar equivalent RC-circuit description of oscillatory membrane properties in barnacle muscle fiber (Morris and Lecar, 1981). These models equate capacitive current (according to Kirchhoff's current law) with the summed active and passive currents across the membrane. Morris–Lecar models can be thought of as reductions of Hodgkin and Huxley's model of the squid axon. They include active currents that describe ligand-gated excitatory (Na^+^) and inhibitory (Cl^−^) ion flow, mediated through fast glutamatergic and GABAergic receptors, with a potassium leak current used to account for all passive ionic currents (Gutkin et al., 2003); where the conductance of the active channels display first order dynamics that depend on the time constant of the channel and their current state (Figure 2).

It may seem that we are conflating the introduction of mean field (versus neural mass) formulations with the introduction of conductance-based models. However, there is a fundamental reason for doing this: in full mean field treatments, the ordinary differential equations describe the dynamics of first and higher-order statistics of population densities—such as the covariance among neuronal states within a population. Crucially, the covariance depends upon the mean of the neuronal states when, and only when, the equations are non-linear in the states (i.e., where the states interact multiplicatively). In other words, the weakly non-linear (sigmoidal) equations of motion of convolution-based models mean that the covariances are not functions of the population averages and therefore do not change with time. This is why one only has to consider first-order statistics in neural mass models based upon (linear) synaptic convolution operators. However, when we move to conductance-based models, there is a necessary interaction between conductance and depolarization, which renders the models intrinsically non-linear. This means that the covariances depend upon the means (and vice versa). It therefore only makes sense to consider mean field treatments of conductance-based models.

Mean field treatments start with a description of a single neuronal response in terms of stochastic differential equations (presented in Figure 2) that accommodate noise or fluctuations in neuronal states (Note: In physics generally a mean-field reduction refers to any statistical summary i.e., to first or higher order, to describe population responses. In the context of neuronal population models of brain function, the literature has a adopted a standard where a neural-mass refers to first order and a mean-field; first and higher order interactions (Deco et al., 2008). Marreiros et al. (2009) used these stochastic differential equations to form a set of non-linear ordinary differential equations by applying the Fokker-Planck formalism using the Laplace assumption (or method of moments). This meant that ensemble activity could be modeled without the need to simulate individual unit activity with noisy fluctuations—the neuronal fluctuations are implicit in the population density dynamics. Heuristically, the population density approach is important because it provides a unique prediction or generative model of an empirical response. Mean field formulations of conductance-based models are therefore invertible (can be fitted to data) in the setting of DCM. The resulting expression for the population dynamics; the evolution of the population's mean and variance, decomposes into deterministic flow and diffusion. In turn, these reduce to simple forms under Gaussian (Laplace) assumptions about the population density—where first order population dynamics are a function of flow and the curvature of the flow, and the second order statistics a function of the gradients of flow. However, a DCM of a single source typically comprises three coupled populations (David and Friston, 2003).

For conductance based models in DCM, we employed a similar structure to the “ERP” and “LFP” mass models—with interacting populations of excitatory spiny stellate cells, pyramidal cells and inhibitory interneurons, coupled through intrinsic connections (Figure 2). The mean field partition means that these operations can be applied to each population in turn, and lead to simple expressions; given that the gradient and curvature of the equations of motion are only non-zero within a particular ensemble. These equations describe a mean-field model; the “MFM,” in which the first and second order sufficient statistics interact, influencing each other when the curvature (derivative of the flow) is non-zero. The formally reduced model, where only first order interactions are considered is termed the “NMM”. This point mass interaction is identical to the ERP and LFP models in terms of the effects different subpopulations exert on others, but have a different dynamic form through the equivalent RC circuit description. Unlike the NMM, the MFM allows for expansion (dynamic increase in the variance of the state's associated probability distribution) and contraction (dynamic reduction in the variance of the state's associated probability distribution) of dispersed neuronal states to influence the average flow.

Marreiros et al. (2010) compared DCM using mean-field models (MFM) with dynamically coupled means and variances to a model where the variance was fixed (NMM). In the time domain, a simulated evoked potential elegantly demonstrated the effect of this coupling with the variance of the pyramidal cell population's depolarization contracting to close to zero when the mean approaches its maximum. Within the spectral domain, they similarly showed qualitative differences in the dynamic repertoire—with the MFM displaying limit-cycle attractors after bifurcation from a fixed-point (Marreiros et al., 2009). In this setting, the mean-field model is inherently more non-linear, because it entails non-linear interactions between the first and second order statistics of the hidden states (i.e., dynamic processes that affect the observations, but cannot be directly measured. the activity in interneurons affect signal propagation within regions but due to the random pattern of their dendrites do not directly contribute to the measured field). This speaks to many similar investigations of non-linearity in neural systems (Lopes Da Silva et al., 1989; Destexhe and Babloyantz, 1991; Daffertshofer et al., 2000; Breakspear, 2002; Breakspear and Terry, 2002; Stam, 2005), where the emergence of quasiperiodic, chaotic and itinerant attractors belies a rich set of dynamic phenomena with physiologically plausible interpretations.

### The NMM and MFM with voltage gated NMDA receptors

An extension to the NMM and MFM was presented in Moran et al. (2011a) through the inclusion of a third ligand-gated ion channel to model conductances controlled by the NMDA receptor. NMDA receptor controlled ion channels were considered in a separate treatment since they are both ligand- and voltage-gated. For an NMDA channel to open, following the binding of glutamate, there must first be a large transmembrane potential to remove a magnesium ion blocking the channel. Hence, the dynamics for this particular current are given by an extended equation, which includes the magnesium component using a voltage gated function (Figure 2). By introducing NMDA ion channels to pyramidal cells and inhibitory interneurons (Brunel and Wang, 2001), we further constrained and distinguished laminar specific responses. This type of channel afforded another source of non-linearity due to its voltage dependency (Jahr and Stevens, 1990), and required an extension of the steady-state linearization, for both the NMM and MFM case.

To characterize the dynamic repertoire of these models, we examined steady-state responses in the frequency domain to identify regimes where the system settles to a fixed point (i.e., the average activity reaches steady-state) or a limit cycle. In the first regime the spectrum observed is generated by noise, where neuronal populations act as a filter—shaping the noise spectrum to produce a profile of output frequencies. In the second dynamic regime, the average neuronal states themselves may oscillate. In this situation, the system exhibits what is known as a quasiperiodic attractor and the frequency response to noise changes with different points on the attractor (Moran et al., 2011a). This means one has to take the average frequency response over the attractor manifold (i.e., over the limit cycle). Crucially, the frequencies that are preferentially passed by the system are also the frequency of the oscillation (limit cycle). This means the predicted spectral responses to noise under steady state can be seen (and treated mathematically) as a special case that obtains when the attractor collapses to a fixed point. This second regime was particularly evident in the MFM case, where attractor subspaces characteristic of heteroclinic channels were observed—and produced bimodal spectral peaks from local and global state space trajectories.

This richly parameterized neural mass model was then used to examine distinctions among the type of receptors underlying empirical neural activations in EEG and MEG. In Moran et al. (2011b), we tried to recover pharmacologically induced changes in receptor processing using MEG, during a visuo-spatial working memory task. Specifically, parameter estimates from the spectral response in superior frontal gyrus, disclosed an effect of L-Dopa on delay period activity—in terms of how L-dopa changed specific synaptic (connectivity) parameters (Figure 2). These effects were exactly commensurate with predictions from the animal and computational literature (Goldman-Rakic, 1996; Durstewitz et al., 2000; Gorelova and Yang, 2000; Gonzalez-Islas and Hablitz, 2003; Durstewitz and Seamans, 2008) and revealed the dual mechanisms of dopaminergic modulation of glutamatergic processing; where L-Dopa increased the non-linearity of post-synaptic responses mediated by NMDA receptors, and decreased AMPA coupling between pyramidal cells and stellate cells. In this study, we also found an L-Dopa-dependent change in exogenous input into the frontal region, which effectively suppresses this region during delay-related reverberatory processing. Moreover, individual parameter estimates from the DCM correlated with individual performance indices (Moran et al., 2011b), a crucial finding that is often used to show that parameter estimates from an NMM reflect real neuronal processes.

## Spatial harmonics and the neural field

### The NFM

Pinotsis et al. (Pinotsis and Friston, 2011; Pinotsis et al., 2012) introduced the spatial domain into DCM with neural field models. Neural fields model current fluxes as continuous processes on the cortical sheet, using PDEs. The key advance that neural field models offer, over conventional neural mass models, is that they embody spatial parameters (like the density and extent of lateral connections). This means that, in principle, one can infer the spatial parameters of cortical infrastructures generating electrophysiological signals (and infer changes in those parameters over different levels of an experimental factor) from empirical data. This rests on modeling responses not just in time but also over space. This sort of model should be ideally suited to exploit the temporal dynamics of observed cortical responses with a high spatial resolution; for example, with high-density recordings, at the epidural or intracortical level. However, as demonstrated in early DCM-NFM, the impact of spatially extensive dynamics is not restricted to expression over space but can also have profound effects on temporal (e.g., spectral) responses at one point (or averaged locally over the cortical surface) (Pinotsis et al., 2012). This means that neural field models may also play a key role in the modeling of non-invasive electrophysiological data that does not resolve spatial activity directly. Although, neural mass models can describe patterns in sensor space, the spatial attributes of these patterns result from the coupling among states at different points in source space and not from hidden states that are functions space (i.e., they are described by equations that are time dependent but not spatially dependent).

In terms of anatomical and physiological constraints, functional specialization demands that cells with common functional properties are grouped together. This architectural constraint necessitates both convergence and divergence of cortical connections (Zeki, 1990), of the sort that can be modeled with a neural field model. To model these spatial aspects of connectivity one needs partial differential or integro differential equations that accommodate lateral interactions over spatially extended cortical manifolds. In Pinotsis and Friston (2011), the repertoire of steady state regimes engendered by sparse (patches of) intrinsic connections was examined. Specifically, we considered a bimodal, non-centric distribution and showed (through a Turing instability analysis) that the dispersion relation from this particular arrangement of spatial delays leads to infinite branches of complex spectra. These branches undergo similar conformational changes, under both increased propagation velocity and decreased spatial separation (range) of lateral connections. The resulting fall in the amplitude of high frequency oscillations was also apparent in the spectral summaries of these responses, in terms of cross spectral densities. For example, as the separation of coupled neuronal populations increases, the total spectral power decreases and falls faster for higher frequencies in a manner similar to local coherence functions based on primate recordings (see Leopold et al., 2003) In brief, both the spatial deployment and the speed of lateral connections can have a profound effect on the behavior of spatial harmonics over different scales. Interestingly, it turned out that only synaptic gain was capable of producing phase-transitions: when increasing gain, the system was driven to an unstable regime and oscillations appeared as a result of a Hopf bifurcation.

**Figure 3:**
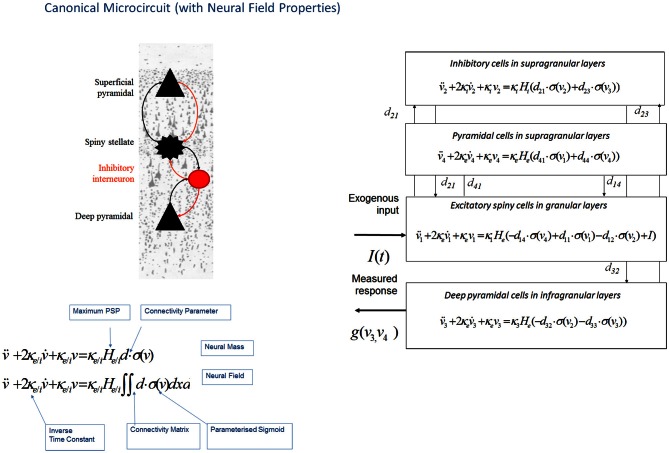
**Canonical microcircuit neural field model: “CMC”.** This figure shows the evolution equations that specify a canonical microcircuit (CMC) neural mass model of a single source. This model contains four populations occupying different cortical layers: the pyramidal cell population of the Jansen and Rit model is effectively split into two subpopulations allowing a separation of the neuronal populations that elaborate forward and backward connections in cortical hierarchies. As with the ERP and LFP models, second-order differential equations (shown earlier in Figure 1 decomposed into two first order ODEs), mediate a linear convolution of presynaptic activity [a sigmoidal function of presynaptic membrane depolarization σ(*v*)] to produce postsynaptic depolarization (*v*), dependent on membrane time constants (*1/*κ_*e*/*i*_) and average post-synaptic depolarizations (*H*_*e*/*i*_) at excitatory (*e*) and inhibitory (*i*) synapses. This depolarization gives rise to firing rates within each sub-population that provide inputs to other populations. Replacing connectivity parameters *d*, with a connectivity matrix over space and time *D(x,t)* enables one to generalize the neural mass model to a neural field model. This effectively converts the ordinary differential equations in this figure into partial differential equations or neural field equations.

This neural field model was later extended to a three layered architecture comprising pyramidal cells, inhibitory interneurons and spiny stellate cells (Figure 3), where spatial delays result from signals propagating with finite conduction speeds along axonal arbors (Pinotsis et al., 2012). These arbors were arranged with a central distribution of synaptic densities, which decayed exponentially in space. Spatial delays operated along the same intrinsic connections used in ERP neural mass models: the cortical micro circuitry in the “ERP” and “NFM” models. This means that the neural mass and field models are essentially the same; describing the same neurobiological dynamics over time, but where the “NFM” is equipped with spatially extended hidden states that characterize presynaptic input as a spatially-extended process that is propagated along axonal arbors. In contrast the hidden states in a neural mass model are a function of time only. As with the “ERP” and “LFP” models, this model (“NFM”) uses a convolution operator to characterize post synaptic filtering and a sigmoid function to accommodate the dispersion of the hidden states of the afferent population (Figure 3).

The advantage of neural field models is that they can accommodate spatially extended activity on cortical manifold or patches that endows the predicted responses with a complicated frequency dependency. This allows one to distinguish between spatial effects and other factors (such as intrinsic cell properties) on the basis of observed (empirical) responses. The incorporation of neural field models in the DCM framework allowed spatial parameters of the sources—like the spatial decay rate of synaptic connections and intrinsic conduction speed—to be optimized even using spatially unresolved data, like a time series from a single LFP channel. For example, Bayesian model selection (BMS) correctly distinguished between mass and field models. This type of comparison was formalized using DCM and Bayesian model evidence in the context of invasive local field potentials from rat auditory cortex. With these invasive data, the neural-field model had a much greater evidence than the equivalent neural mass variant; this could be attributed to the increased repertoire of predictions that these models afford and indicates a key role for—and parameterization of—spatial as well as temporal dynamics on the cortical manifold.

### Structure, function, and the canonical microcircuit (CMC)

Pinotsis et al. (2013b) showed that DCM with neural fields can provide a detailed analysis of correlations between cortical structure and function. This analysis was motivated by previous results suggesting two hypotheses regarding the biophysical basis of inter-individual differences in peak gamma frequencies—one based on functional differences and one based on structural differences: Muthukumaraswamy et al. (2009) suggested that peak gamma frequency is determined by the level of inhibition in V1, as described by resting GABA concentration measured with MR spectroscopy. Later, Schwarzkopf et al. (2012) found a correlation between peak gamma frequency and the surface area of primary visual cortex as measured with retinotopic mapping. These authors suggested that the size of V1 and associated differences in structural microanatomy could be true determinants of peak gamma frequency. The above two hypotheses suggest that both GABA concentration and V1 size can influence gamma frequency; however, these factors may or may not be causally linked. In other words, a larger V1 may have a higher GABA concentration that may or may not be due to a higher local GABA density.

DCM with neural fields incorporate parameters pertaining to both microanatomy and the density of GABA receptors (that determine inhibitory intrinsic connection strengths) and allow one to investigate alternative explanations for differences in gamma peak frequency. These differences could be mediated by either a kinetic (functional) parameter, summarizing the level of cortical inhibition or the (structural) macrocolumn width or both of these parameters. Pinotsis et al. (2013b) looked at the correlations over subjects between peak gamma frequency, V1 surface area and the Bayesian estimates of these structural and functional parameters and found that both hypotheses were confirmed. In brief, they found correlations between columnar width, gamma peak and V1 size and also between the GABAergic parameter and the gamma peak. This correlation remained significant when controlling for V1 size and width. This suggests that the correlation between gamma peak and V1 inhibition cannot be accounted for completely by the spatial parameters (at the microscopic or macroscopic level). Structural equation modeling was used to characterize the causal dependencies among observed quantities and the model parameters: in the winning structure equation model, peak gamma frequency was mediated proximately by excitatory drive to inhibitory (GABAergic) interneurons and the strength of this drive was determined, in part, by the size of macrocolumns. In turn, the size of the macrocolumn was constrained by the macroscopic (retinotopic) size of V1 (under the assumption that V1 size is determined genetically or epigenetically). These results suggested that both cortical microstructure and excitability may be important for visual perception and are in accord with empirical studies showing that the size of V1 is negatively correlated with the strength of visual illusions (Schwarzkopf et al., 2010)—and that GABA concentration correlates with orientation discrimination ability (Edden et al., 2009).

This work also introduced an expanded neuronal architecture based upon the canonical microcircuit. These models comprise four subpopulations (as opposed to three subpopulations in mass and field models above, see Figure 3). The canonical microcircuit or “CMC” models a refinement of the Jansen and Rit convolution models that explicitly accommodates the neuronal sources of forward and backward connections in cortical hierarchies (Bastos et al., 2012). These are distinct superficial and deep pyramidal cell populations, respectively that, crucially, may exhibit different spectral outputs. Specifically gamma responses have been recorded in superficial layers, while slower dynamics (in the alpha and beta range) arise concurrently in infra granular populations (Maier et al., 2010; Buffalo et al., 2011; Bastos et al., 2012). The CMC proposes an intrinsic connectivity architecture to account for this non-linear transformation though dendritic and population effects (Bastos et al., 2012). The canonical microcircuit model is based upon intracellular recordings in cat visual cortex by Douglas and Martin (1991) who investigated the laminar propagation of afferent signals and produced pathways that are thought to reflect canonical input–output processing streams for forward and backward signals throughout the cortex. These models are currently being used to test hypotheses about asymmetries in forward and backward message passing that may shed light on the distributed neural processing that underlies perceptual synthesis and inference.

## Summary

The basic idea behind DCM is that neural activity propagates through brain networks in a way that can be modeled as an input-state-output-system, where causal interactions within the system are mediated by unobservable (hidden) dynamics. The resulting multi-input-multi-output (MIMO) neuronal model is then supplemented with a observation model (e.g., classical electromagnetic forward model) that describes the mapping from neural activity to observed responses (Daunizeau et al., 2011). It is the dynamics of the hidden states that are prescribed by the neural mass and neural field models outlined above. The type of data and data features determine the combination of neuronal and observation models. For example, EEG and MEG data require a different observation model than LFP data, while evoked responses necessitate a parameterized Gaussian pulse input—in contrast with spectral density data features that require parameterized neuronal noise spectra. In all of these different applications, the underlying “LFP,” “ERP,” “NMM,” “MFM,” “NFM,” and “CMC” models are, in principle, interchangeable.

The choice of the appropriate neuronal model should reflect the research question at hand: for example, whether the focus is on topographic as opposed to intrinsic neurotransmitter properties or drug effects etc. This choice may also be informed by previous applications, where a particular model has already proven useful within a DCM context (for example, fast oscillations in the gamma band for the “CMC” model). One generally designs a DCM study to assess the effects of task manipulation, group, pathology or drug on a particular parameter or set of parameters of interest. In other words, the choice of model should be evident at the outset and often conforms to the “minimal model approach” necessary to access that parameter—this is because, in general, a simpler model has more constraints and can use the degrees of freedom in the data to estimate model parameters and evidence more efficiently. For example, where differences in effective extrinsic connectivity are of interest, a convolution based model—that is agnostic to specific intrinsic ion channel mediators—will suffice to address the hypothesis (Campo et al., 2012). The direction of empirical research using DCM as a “mathematical microscope” of synaptic processes—where particular receptor and neurotransmitter changes are important—may call for the finer grained physiological details of the NMM or MFM (Moran et al., 2011b). In other cases the form of the dynamics, for example whether field (or propagation) effects should be considered, may itself embody the central hypothesis. In this case, as demonstrated in Pinotsis et al. (2013b), BMS may be sufficient to disambiguate among competing hypotheses about neuronal architecture (Penny et al., 2004). In effect, BMS usurps all other arguments as the best method to test which model should be applied to which data; though computational and time constraints, particularly as the suite of options in DCM is expanded, may determine the extensiveness and overall feasibility of such a search. In principle, researchers may employ their own neuronal model (or feature extraction process) by compositing a parameterized state space and utilizing the modularity of SPM's source code. Wrapper routines which specify parameter priors, integration schemes and variational expectation maximization can be applied to a function of neuronal activity that is user-specified (In SPM's DCM toolbox: http://www.fil.ion.ucl.ac.uk/spm/, The current routines are described in files “spm_fx_nmm”) (Kiebel et al., 2009; Litvak et al., 2011). Construct validity would then need to be tested by simulating data from different regions of parameter space and investigating parameter identify ability i.e., whether the framework can recover the simulated parameters given different initializations. These tests are particularly important in the case of highly nonlinear state space models, given the potential of the gradient ascent to converge to local maxima (Friston et al., 2012).

The models we have reviewed in this paper may also be useful beyond the DCM inference framework. For example, in the study of large scale generative processes underlying resting-state networks in fMRI, neural mass (Deco and Jirsa, 2012) and neural field models (Pinotsis et al., 2013a) have been embedded in anatomical graphs to study emergent behaviors and dynamic properties (Gray et al., 2009; Robinson et al., 2009). This suggests that these models may have some utility as neural state equations in DCM for fMRI, though currently we deploy coarser models with far less physiological detail (Daunizeau et al., 2011). The field of inquiry using these types of models is varied and rich, with DCM applications including the locus of consciousness and unconsciousness in vegetative state patients (Boly et al., 2011), diaschisis in temporal lobe epilepsy (Campo et al., 2012) and the effects of ketamine on synaptic plasticity (Schmidt et al., 2012). The set of distinct dynamics within which these types of effects can be parameterized will no doubt grow beyond the current suite of models available. Indeed their exchangeability within the DCM framework allows researchers to define their own favorite or interesting model and proceed in the usual Bayesian way (Friston et al., 2003).

### Conflict of interest statement

The authors declare that the research was conducted in the absence of any commercial or financial relationships that could be construed as a potential conflict of interest.
